# Gene expression profile analysis to discover molecular signatures for early diagnosis and therapies of triple-negative breast cancer

**DOI:** 10.3389/fmolb.2022.1049741

**Published:** 2022-12-07

**Authors:** Md Shahin Alam, Adiba Sultana, Guanghui Wang, Md Nurul Haque Mollah

**Affiliations:** ^1^ Laboratory of Molecular Neuropathology, Department of Pharmacology, Jiangsu Key Laboratory of Neuropsychiatric Diseases and College of Pharmaceutical Sciences, Soochow University, Suzhou, Jiangsu, China; ^**2**^ Bioinformatics Lab. (Dry), Department of Statistics, University of Rajshahi, Rajshahi, Bangladesh

**Keywords:** triple-negative breast cancer, microarray gene expression profiles, key genes (KGs), diagnosis, therapies, integrated bioinformatics and system biology approaches

## Abstract

Triple-negative breast cancer (TNBC) is one of the most lethal subtypes of breast cancer (BC), and it accounts for approximately 10%–20% of all invasive BCs diagnosed worldwide. The survival rate of TNBC in stages III and IV is very low, and a large number of patients are diagnosed in these stages. Therefore, the purpose of this study was to identify TNBC-causing molecular signatures and anti-TNBC drug agents for early diagnosis and therapies. Five microarray datasets that contained 304 TNBC and 109 control samples were collected from the Gene Expression Omnibus (GEO) database, and RNA-Seq data with 116 tumor and 124 normal samples were collected from TCGA database to identify differentially expressed genes (DEGs) between TNBC and control samples. A total of 64 DEGs were identified, of which 29 were upregulated and 35 were downregulated, by using the statistical limma R-package. Among them, seven key genes (KGs) were commonly selected from microarray and RNA-Seq data based on the high degree of connectivity through PPI (protein–protein interaction) and module analysis. Out of these seven KGs, six KGs (TOP2A, BIRC5, AURKB, ACTB, ASPM, and BUB1B) were upregulated and one (EGFR) was downregulated. We also investigated their differential expression patterns with different subtypes and progression stages of BC by the independent datasets of RNA-seq profiles from UALCAN database, which indicated that they may be potential biomarkers for early diagnosis. Gene Ontology (GO) and the Kyoto Encyclopedia of Genes and Genomes (KEGG) pathway enrichment analyses with the proposed DEGs were performed using the online Enrichr database to investigate the pathogenetic processes of TNBC highlighting KGs. Then, we performed gene regulatory network analysis and identified three transcriptional (SOX2, E2F4, and KDM5B) and three post-transcriptional (hsa-mir-1-3p, hsa-mir-124-3p, and hsa-mir-34a-5p) regulators of KGs. Finally, we proposed five KG-guided repurposable drug molecules (imatinib, regorafenib, pazopanib, teniposide, and dexrazoxane) for TNBC through network pharmacology and molecular docking analyses. These drug molecules also showed significant binding performance with some cancer-related PTM-sites (phosphorylation, succinylation, and ubiquitination) of top-ranked four key proteins (EGFR, AURKB, BIRC5, and TOP2A). Therefore, the findings of this computational study may play a vital role in early diagnosis and therapies against TNBC by wet-lab validation.

## Introduction

Breast cancer (BC) is a major public health concern as it is one of the most common types of hormone-sensitive cancers and the most commonly diagnosed cancer in women worldwide. GLOBOCAN estimates approximately 2,261,419 (11.7% of all cancers, the most diagnosed cancer type) newly diagnosed cases, and 684,996 (6.9% of all cancers) deaths were reported due to BC in 2020 (Sung et al., 2021). There are four major molecular subtypes of BC, namely, luminal A, luminal B, HER2, and triple negative/basal-like (Yanagawa et al., 2012). TNBC is a specific subtype of BC that is estrogen receptor-negative (ER-), progesterone receptor-negative (PR-), and HER2-negative (HER2-). Although approximately 10–20% of BC is covered by TNBC, it has a higher mortality, is metastatic, and more aggressive compared to other subtypes (Kumar and Aggarwal, 2016). Although there have been significant advances in systematic treatment for BC over the decades, TNBC has not benefited from advanced treatment strategies due to the lack of TNBC-specific therapeutic targets. The 5-year overall survival rate for patients with TNBC is lower than that of other subtypes of BC (Dent et al., 2007). In particular, the 5-year overall survival rate is 91% if the cancer is diagnosed at an early stage, it drops to 65% if the cancer spreads to the lymph nodes near the breast, and eventually it drops to a very low 12% if the cancer spreads to distant sites (Society, 2022). Therefore, it is important to identify biomolecular signatures that can diagnose TNBC at an early stage and play a vital role as therapeutic targets in treatment strategies to reduce mortality in TNBC patients. The TNBC-driving genes may play the key role in this regard. There are some transcriptomics studies exploring TNBC-driving key genes (KGs). However, we observed that their individual KG-sets were not so consistent (see Supplementary Table S1 in Supplementary File S1). It may have happened due to the environmental variations, small sample sizes, and selection of inappropriate statistical models. They did not validate their suggested KGs against the other independent databases. Also, they did not explore their suggested KG-guided candidate drug molecules for the treatment against TNBC. Therefore, the main purpose of this study was to explore more consistent TNBC-driving KGs for early diagnosis and therapies, giving the weight to a large sample size, appropriate statistical model, and the datasets from different environment.

**FIGURE 1:**
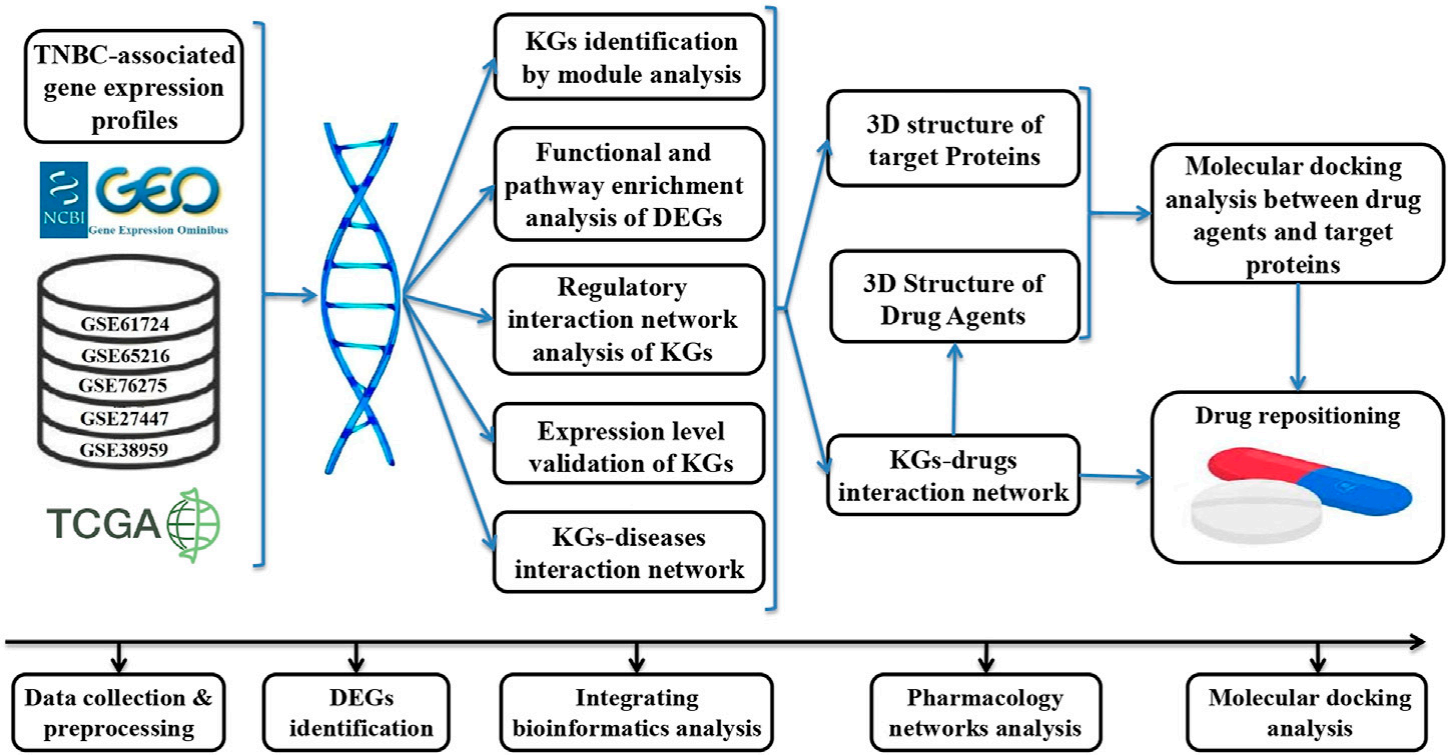
Workflow of this study.

Transcriptomics analysis is one of the most popular platforms for identifying disease-driving genes (Li et al., 2021; Yuan et al., 2021; Alam et al., 2022a; Alam et al., 2022b; Alam et al., 2022c; Ma et al., 2022). In this study, we created transcriptomics datasets of large sample sizes from NCBI-GEO and TCGA databases to identify more consistent TNBC-driving KGs for diagnosis at an early-stage. To make a large sample size, we combined five environmentally independent microarray gene-expression datasets from the NCBI-GEO database that contained 304 TNBC and 109 control samples in total. On the other hand, the RNA-Seq profiles from TCGA database consisted of 116 TNBC and 124 control samples that were generated from different environments. In the case of drug discovery, drug repositioning (DR) is a promising strategy for discovering new therapies through existing drugs (Langedijk et al., 2015). The DR strategy is efficient, safe, less expensive, and less time-consuming than the *de novo* technique (Rudrapal et al., 2020). Therefore, in this study, we also attempted to explore KG-guided repurposable drug-molecules through the network pharmacology and molecular docking analysis. Network pharmacology is an efficient tool to generate interactions between therapeutic targets (genes) and drug molecules through a network-based approach (Hopkins, 2007; Song et al., 2019). Recently, molecular docking analysis has gained popularity in the field of computational research to identify therapeutic target-related candidate drugs (Alam et al., 2022a; Alam et al., 2022b). In this study, we considered well-established bioinformatics tools and statistical methods to identify biomolecular signatures and candidate drug agents that may play an effective role in the early diagnosis and treatment of TNBC patients. The whole workflow of this study is displayed in Figure 1.

## Methods and materials

### Data collection

A total of 304 TNBC samples and 109 normal samples were collected from five sets of omics data (gene expression profiles data) with accession numbers GSE65216 (Dubois, 2019), GSE76275 (Fuqua, 2019), GSE38959 (Komatsu and Katagiri, 2018), GSE27447 (Yang et al., 2018), and GSE61724 (Mathe et al., 2018), and 116 tumor samples and 124 normal samples were collected from RNA-Seq data. Datasets were downloaded from the public database Gene Expression Omnibus (GEO) in the National Center of Biotechnology Information (NCBI) (Edgar and Barrett, 2006) and TCGA database (https://www.cancer.gov/about-nci/organization/ccg/research/structural-genomics/tcga). Two datasets (GSE65216 and GSE76275) are based on the platform GPL570 Affymetrix Human Genome U133 Plus 2.0 Array, two datasets (GSE27447 and GSE61724) are based on the platform GPL6244 Affymetrix Human Gene 1.0 ST Array, and the last one (GSE38959) is based on the platform GPL4133 Agilent-014850 Whole Human Genome Microarray. More details of the datasets are given in Table 1.

**TABLE 1:** List of datasets with brief descriptions that are used in this study.

Accession number	Sample size		Probe	Platform	Country
	Tumor	Normal			
**GSE65216**	55	11	54,673	GPL570 Affymetrix Human Genome U133 Plus 2.0 Array	France
**GSE76275**	198	67	54,675		United States
**GSE38959**	30	13	45,015	GPL4133 Agilent-014850 Whole Human Genome Microarray 4 × 44K G4112F	Japan
**GSE27447**	5	14	33,297	GPL6244 Affymetrix Human Gene 1.0 ST Array	United States
**GSE61724**	16	4	33,297		Australia
**Total**	304	109			
**TCGA data**	116	124	49,449	NA	NA

### Data preprocessing and DEG identification

Background correction, standardization, and median polish summarization were performed on the raw data through the robust multichip average (RMA) algorithm analysis (Irizarry et al., 2003). Then, we converted the probe IDs to official gene symbols according to the annotation files and merge all datasets. After combining the five datasets, we obtained a total number of samples of 413 including 304 TNBC and 109 normal. We considered the limma (Linear Models for Microarray Analysis) statistical approach (Ritchie et al., 2015), which was implemented in the limma (v- 3.44.3) R-package to identify differentially expressed genes (DEGs) between tumor and normal samples. The moderated t-statistic was used to test the null hypothesis (H_0_) (equally expressed gene (EEG) in both tumor and control groups) *versus* the alternative hypothesis (H_1_) (differentially expressed gene (DEG) between tumor and control groups). We have considered the threshold to identify the up- and down-regulated DEGs for the combined data as follows:

$${\mathrm{DEG}}_{g}=\left\{\begin{array}{cc} \mathrm{DEG}\;\left(Upregulated\right), & if\;adj.p.value<0.01\;and\;L{og}_{2}FC>+1.0\\ \mathrm{DEG}\;\left(Downregulated\right), & if\;adj.p.value<0.01\\ & \;and\;L{og}_{2}FC<-1.0 \end{array}\right.,$$

where adj. *p*-value: adjusted *p*-values and Log_2_FC: Log_2_ (fold change).

### PPI network and module analysis

We used the online database Search Tool for the Retrieval of Interacting Genes (STRING) to construct the PPI network of DEGs and visualized it using Cytoscape software (Shannon et al., 2003; Szklarczyk et al., 2019). Module analysis was performed using the MCODE app in Cytoscape with the threshold degree = 2, haircut cluster, node_score = 0.2, k-core = 2, and max. depth = 100.

The association of KGs with different subtypes and progression stages of BC.

We investigated the association of KGs with different subtypes and progression stages of BC using a box plot of their independent RNA-Seq profiles from the UALCAN online database (http://gepia.cancer-pku.cn/index.html) (Chandrashekar et al., 2022). This RNA-Seq dataset contained a total of 114 normal samples and 719 BC samples (luminal = 566, HER2-positive = 37, and triple-negative = 116). On the other hand, the samples sizes are 183, 615, 247, and 20 in stage 1, stage 2, stage 3, and stage 4, respectively.

### GO and KEGG enrichment analysis of DEGs

Gene Ontology (GO) analysis was performed to define and describe genes across species in three categories including biological process (BP), cellular component (CC), and molecular function (MF). The Kyoto Encyclopedia of Genes and Genomes (KEGG) is the databases of drugs, genomes, biological pathways, enzymes *etc.* GO functional and KEGG pathway enrichment analyses were performed using the online tool Enrichr (Chen et al., 2013).

### Regulatory interaction network of KGs

We performed the regulatory interaction network (TF-KG-miRNA) to identify the transcriptional and post-transcriptional regulators of KGs. The regulatory interaction network was constructed using the online tool “NetworkAnalyst” (version: 3.0) (Zhou et al., 2019). The ChEA database was selected for constructing a KG-TF interaction network and miRTarBase database for a KG-miRNA interaction network.

### Drug repositioning

To propose candidate drug molecules for treatment against TNBC, we performed network pharmacology and molecular docking analyses. We considered our proposed KGs as drug target receptor proteins. KG-guided drug agents were detected by the Drug–Gene Interaction Database (DGIdb) through network pharmacology analysis (Wagner et al., 2016). Molecular docking analysis was performed to examine structural binding between receptor proteins and drug agents. We collected 3-D (three-dimensional) structures of both protein receptors and drug agents for molecular docking analysis. We downloaded the 3D structures of all receptor proteins such as TOP2A, BIRC5, EGFR, and AURKB with PDB IDs 1zxm, 1xox, 3GKW, and 4af3, respectively, from the Protein Data Bank (PDB) database (Berman et al., 2000). The 3D structures of drug agents were downloaded from the PubChem database (Kim et al., 2019). Pymol was used to preprocess the 3D structures of ligands, compounds, and the water molecules, and co-crystal ligands which were bound to the protein were removed (DeLano and Bromberg, 2004). We performed molecular docking analysis between the proposed receptors and drug molecules to calculate binding affinity scores (kcal/mol) through the AutoDock Vina in PyRx software (Trott and Olson, 2010; Dallakyan and Olson, 2015). The 3D and 2D structures of interaction between the proposed receptors and top-ranked drug molecules were constructed and visualized using USCF Chimera program and Discovery Studio Visualizer 2021 software (Pettersen et al., 2004). Then, we validated the selected drug molecules with the cancer-related post-translational modification (PTM) sites (phosphorylation, ubiquitination, and succinylation) through docking analysis (Hasan et al., 2016; Wang et al., 2020; Holstein et al., 2021; Mu et al., 2021).

## Result

### Identification of DEGs

**FIGURE 2:**
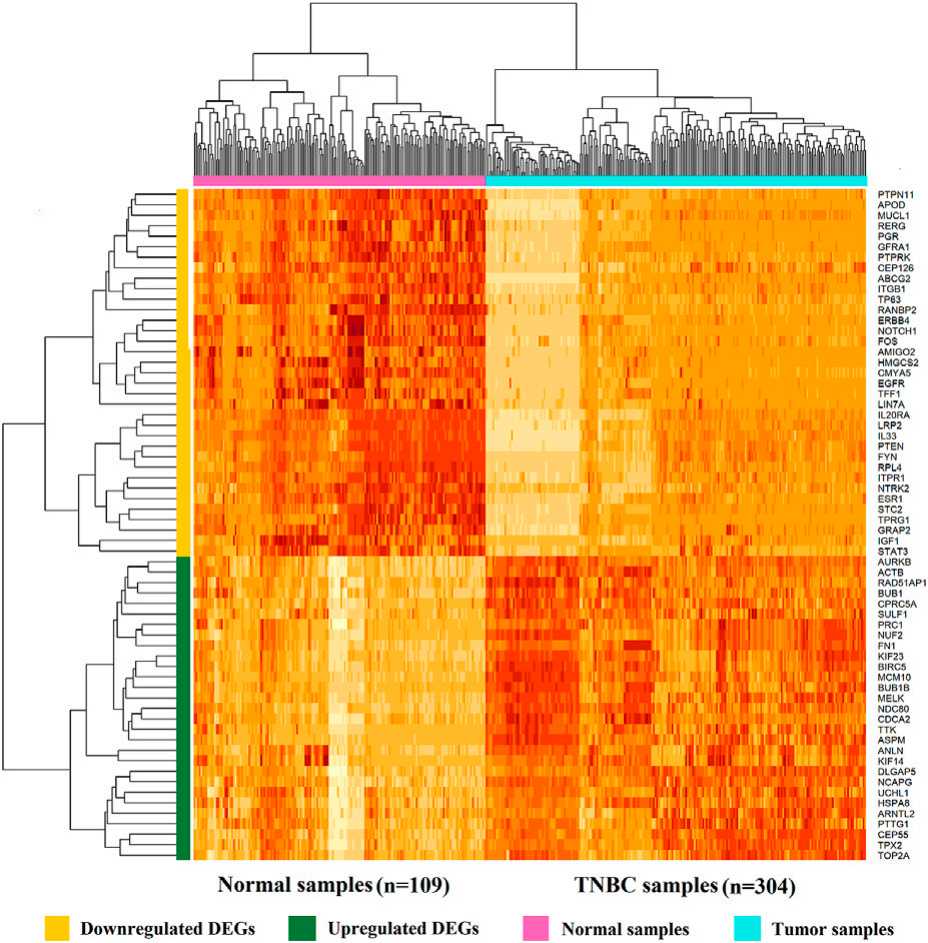
Heatmap of 64 identified DEGs based on integrated microarray analysis of five datasets. Each row represents a single gene, and each column represents a tissue (sample).

We identified a total of 64 DEGs, including 29 up- and 35 down-regulated DEGs between 304 TNBC samples and 109 normal samples in Supplementary File S2 **,** and 306 DEGs including 68 upregulated and 238 downregulated for RNA-Seq data in Supplementary File S3. We visualize the heatmap of hierarchical clustering (HC) for 64 DEGs in Figure 2, where each row represents a single gene and each column represents a sample. We observed that the heatmap clearly classified upregulated and downregulated DEGs, and also tumor and normal samples, where yellow, green, pink, and turquoise indicated downregulated DEGs, upregulated DEGs, normal samples, and tumor samples, respectively.

### Identification of key genes (KGs) by PPI network and cluster analysis

**FIGURE 3:**
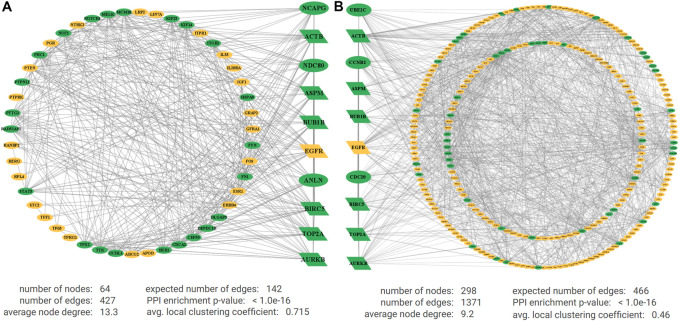
Protein–protein interaction (PPI) networks for **(A)** DEGs for microarray data and **(B)** DEGs for RNA-Seq data. The green indicates upregulated and yellow indicates downregulated DEGs, and the larger nodes in the center are KGs with the highest degree of connectivity.

The PPI network of DEGs for NCBI data included 64 nodes, 427 edges, average node degree of 13.3, and PPI enrichment *p*-value < 1.0e-16. The PPI network of DEGs for TCGA data included 298 nodes, 1,371 edges, average node degree 9.2, and PPI enrichment *p*-value < 1.0e-16. Figure 3A represents the interactions between oncogenes and tumor suppressor genes detected from five NCBI gene-expression profiles, and Figure 3B represents the interactions between oncogenes and tumor suppressor genes detected from TCGA RNA-Seq profiles, where yellow indicates downregulated (tumor suppressor genes), green indicates upregulated (oncogenes), and the parallelogram indicates the common KGs. Then, we selected 10 top-ranked Hub-DEGs for each of NCBI and TCGA datasets. Then, we found seven common Hub-DEGs (TOP2A, BIRC5, AURKB, ACTB, ASPM, BUB1B, and EGFR) between two independent sets of 10 top-ranked Hub-DEGs (see Figures 3A,B). Then, we performed their module analysis and observed that the PPI network of NCBI data produces two modules between oncogenes and tumor suppressor genes with around a 6% clustering error rate including 10 Hub-DEGs with the highest degrees. Furthermore, we found only two modules of DEGs for NCBI data with scores 23 and 12.7. Also, we found a total of 12 modules of DEGs for TCGA data with scores ranging from 31 to 3 through MCODE analysis, and two top-ranked modules for both data are presented in Supplementary Figures S1A,B in Supplementary File S1. Finally, six common upregulated KGs (BIRC5, TOP2A, ASPM, ACTB, BUB1B, and AURKB) and one common downregulated KG (EGFR) were found in module 1 and module 2, respectively, with the highest degree of connectivity for both NCBI and TCGA data in Table 2. The original results of the aforementioned analysis are presented in Supplementary Files S4,S5.

**TABLE 2:** Similarity and consistency of different computational results with KGs.

KGs	Results with NCBI data					
	Fold change (FC)		Limma	PPI	MCODE	
	Log_2_FC	Regulation	adj *p*-value	Degree	Clustering	Degree
TOP2A	2.86	Up	3.80E-07	28	Cluster 1	22
BIRC5	1.87	Up	0.00012	31	Cluster 1	23
EGFR	−1.96	Down	1.61E-05	27	Cluster 2	10
AURKB	1.53	Up	0.001892	25	Cluster 1	23
ACTB	1.78	Up	7.54E-03	24	Cluster 1	21
ASPM	1.95	Up	0.0025	24	Cluster 1	21
BUB1B	1.58	Up	3.80E-04	23	Cluster 1	21
	**Results with TCGA data**					
TOP2A	2.8	Up	1.96E-94	35	Cluster 1	31
BIRC5	2.4	Up	7.78E-85	34	Cluster 1	30
EGFR	−2.3	Down	1.19E-80	45	Cluster 2	16
AURKB	2.8	Up	2.07E-62	35	Cluster 1	31
ACTB	2.0	Up	8.82E-81	70	Cluster 1	31
ASPM	1.8	Up	1.64E-61	35	Cluster 1	31
BUB1B	1.5	Up	1.48E-68	34	Cluster 1	31

**FIGURE 4:**
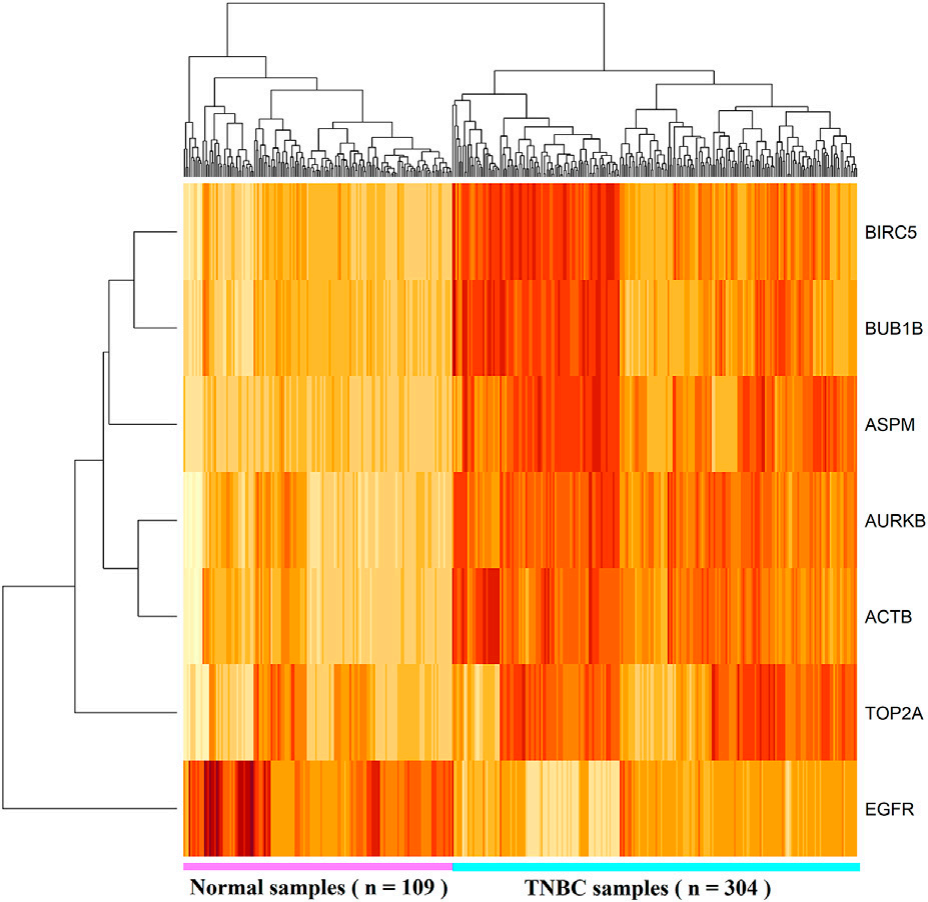
Heatmap of hierarchical clustering (HC) results based on the expressions of seven KGs with 304 TNBC and 109 control samples (dependent data).

To investigate the similarity and consistency of different computational results with KGs, we summarized them in Table-2. We observed that limma and an FC recommended cluster with the upregulated KGs (TOP2A, BIRC5, and AURKB) and the cluster with downregulated KGs (EGFR) are supported by the PPI and MCODE-mediated clusters 1 and 2, respectively. Evidently, results are consistent and support each other. To validate the differential expression patterns of the identified KGs, we considered both dependent and independent test-datasets. Figure 4 shows the HC results based on the expressions of seven KGs with 304 TNBC and 109 control samples (dependent data). We observed that HC clearly separated KGs into upregulated and downregulated groups, and samples/patients in TNBC and control groups.

### Association of KGs with different subtypes and progression stages of BC

**FIGURE 5:**
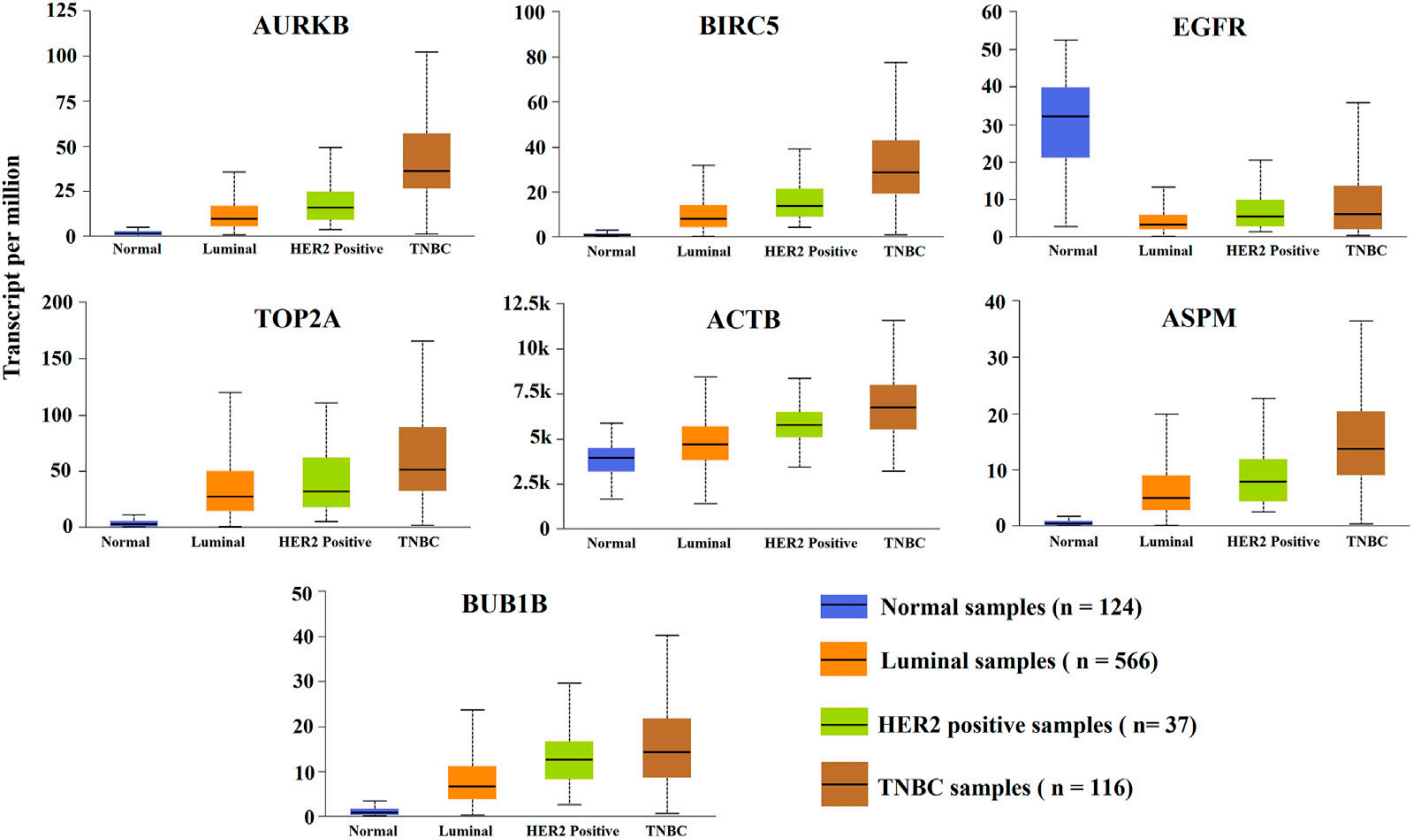
Expression levels’ analysis of KGs between the normal group and the three major subtypes of BC (luminal, HER2-positive, and triple-negative BC).

To investigate the association of KGs (AURKB, BIRC5, TOP2A, ACTB, ASPM, EGFR, and BUB1B) with different subtypes and progression stages of BC by the independent datasets, we performed box plot analysis based on independent RNA-Seq profiles from the UALCAN online database. Figure 5 displayed the box plots for the expressions of KGs with each BC subtypes (normal, luminal, HER2-positive, and TNBC), where blue indicates the normal group and orange, green, and brown indicate luminal, HER2-positive, and triple-negative BC, respectively. We observed the significant differential expression patterns between the normal group and the subtypes of the BC group. Supplementary Figure S2 shows that the expression patterns of KGs with different BC progression stages significantly differentiated from the control group. Therefore, proposed KGs (AURKB, BIRC5, TOP2A, ACTB, ASPM, EGFR, and BUB1B) may play a significant role for the diagnosis of TNBC at the earlier stage.

### GO terms and KEGG pathway enrichment analysis of DEGs

**FIGURE 6:**
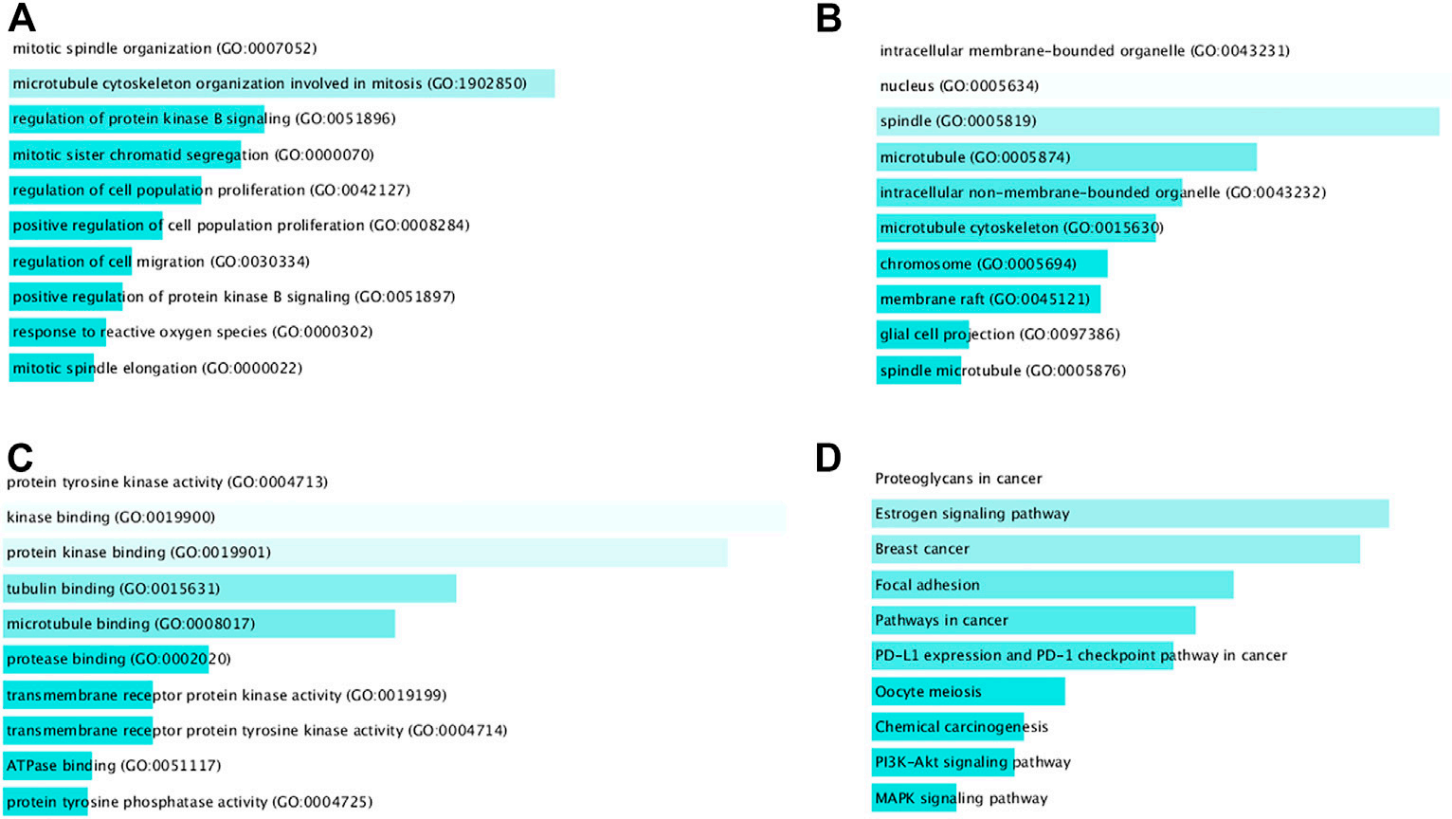
Top 10 significantly enriched terms for each category of DEGs. **(A)** GO BP-terms, **(B)** GO CC-terms, **(C)** GO MF-terms, and **(D)** KEGG pathways.

GO and KEGG enrichment analysis showed that our identified DEGs were enriched by 63 BP-terms, 14 CC-terms, 19 MF-terms, and 48 KEGG-terms, and the threshold *p* < 0.01 was considered. The top 10 significantly enriched terms for each category are presented in Figures 6A–D. We observed that mitotic spindle organization (*p* = 2.4E-15, DEGs involved = 25%), microtubule cytoskeleton organization involved in mitosis (*p* = 9.1E-12, DEGs involved = 21%), intracellular membrane-bound organelle (*p* = 6.8E-08, DEGs involved = 58%), nucleus (*p* = 6.3E-08, DEGs involved = 53%), kinase binding (*p* = 1.9E-06, DEGs involved = 18%), and protein kinase binding (*p* = 4.3E-06, DEGs involved = 22%) were significantly enriched GO terms in TNBC in Figures 6A–C. Proteoglycans in cancer (*p* = 9.6E-10, DEGs involved = 21%), estrogen signaling pathway (*p* = 2.7E-07, DEGs involved = 27%), and pathways in cancer (*p* = 6.7E-06, DEGs involved = 15%) were significantly enriched KEGG pathways terms in Figure 6D. We included further information of GO and KEGG analysis in Supplementary Table S2 in Supplementary File S1.

### Regulatory interaction network of KGs

**FIGURE 7:**
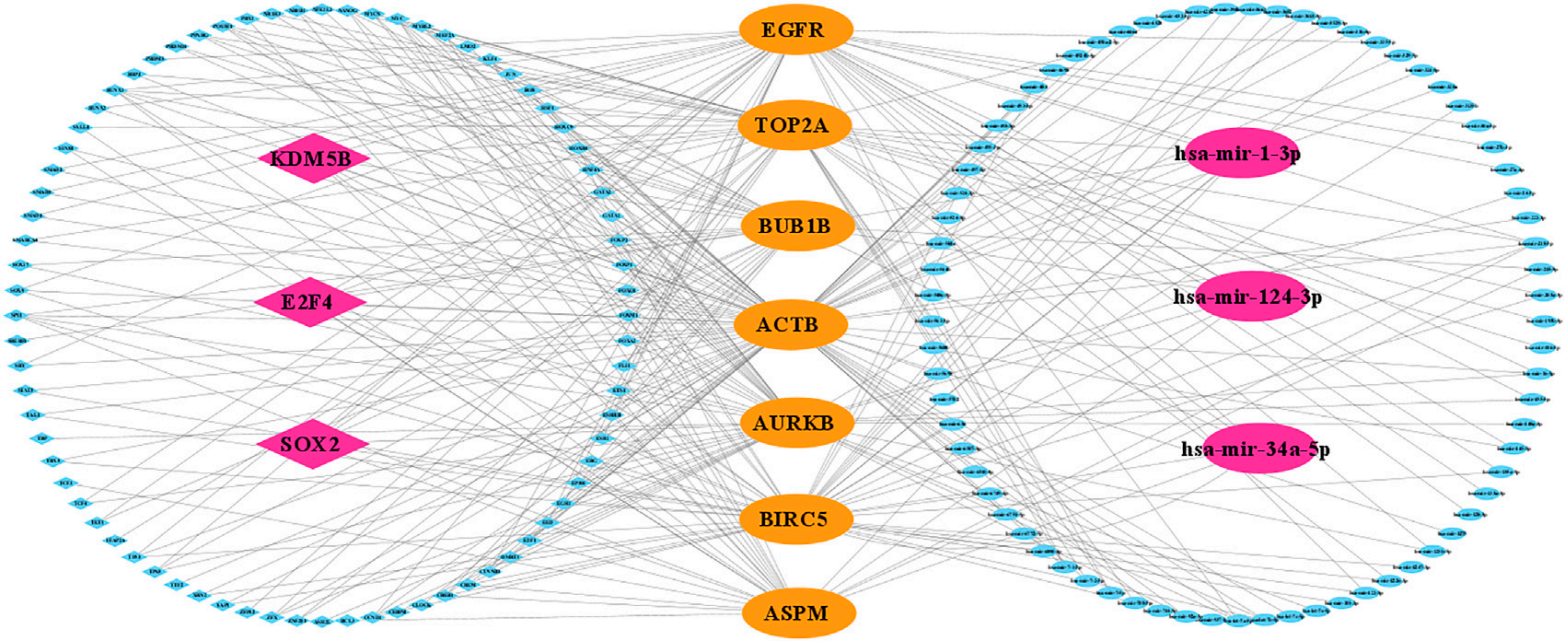
KG-regulatory (TF and miRNA) interaction network, where yellow indicates KGs and pink indicates key TFs (diamond) and key miRNAs (ellipse).

The regulatory interaction network (TF-KG-miRNA) is shown in Figure 7; where pink indicates TFs (diamond) and miRNAs (ellipse) and yellow indicates KGs. Then, we observed that three TFs (SOX2, E2F4, and KDM5B) and three miRNAs (hsa-mir-1-3p, hsa-mir-124-3p, and hsa-mir-34a-5p) are connected with all KGs (degree = 7). Thus, we considered these three TFs and three miRNAs as transcriptional and post-transcriptional factors of KGs.

### Drug repositioning

**FIGURE 8:**
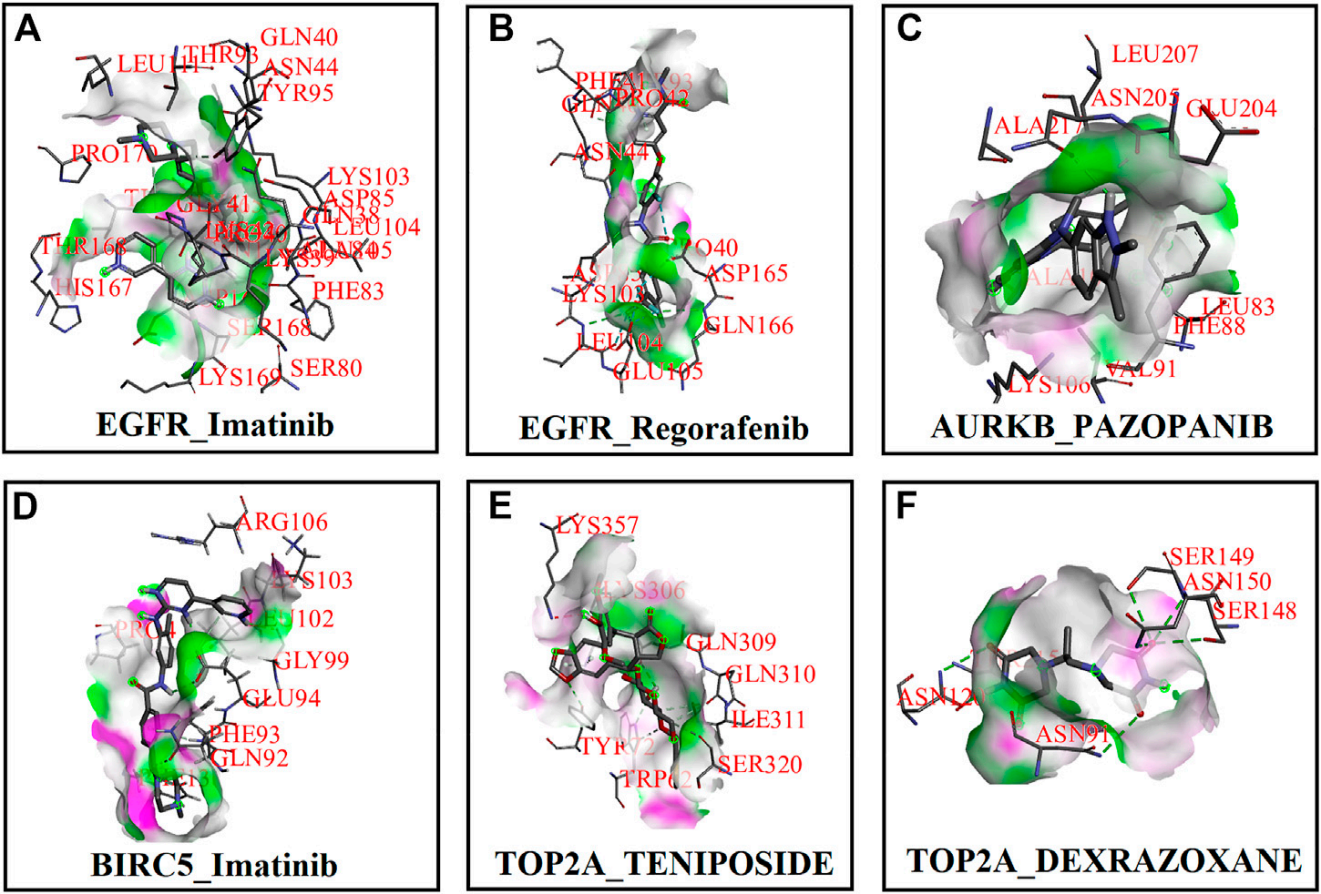
Three-dimensional (3D) structures between receptor proteins and candidate drugs and highlighting active site. **(A)** EGFR–imatinib, **(B)** EGFR–regorafenib, **(C)** AURKB–pazopanib, **(D)** BIRC5–imatinib, **(E)** TOP2A–teniposide, and **(F)** TOP2A–dexrazoxane.

**FIGURE 9:**
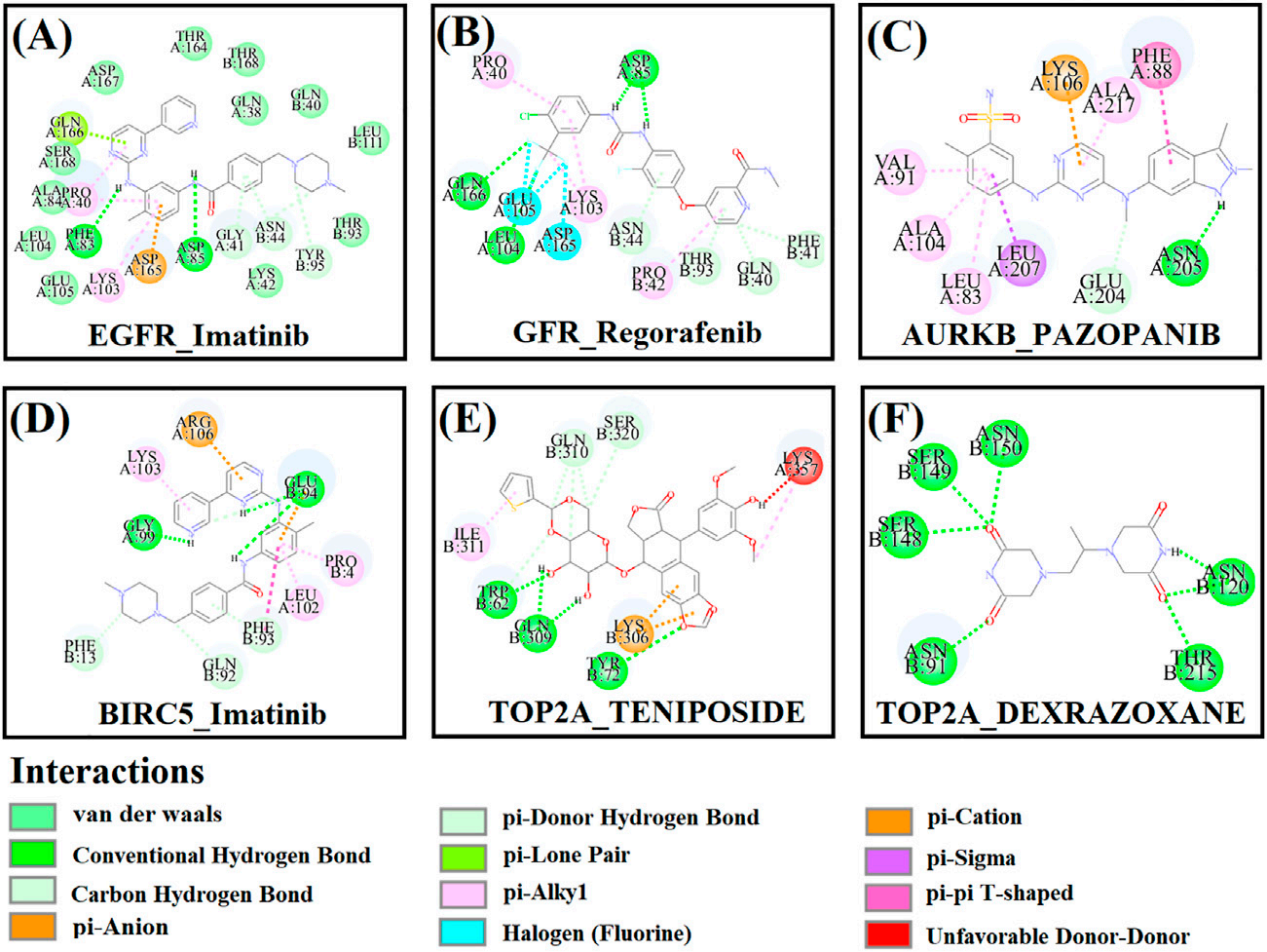
Two-dimensional (2D) structures between receptor proteins and candidate drugs and highlighting interaction types. **(A)** EGFR–imatinib, **(B)** EGFR–regorafenib, **(C)** AURKB–pazopanib, **(D)** BIRC5–imatinib, **(E)** TOP2A–teniposide, and **(F)** TOP2A–dexrazoxane.

First, we collected a total of 115 FDA-approved drugs related to KGs from the DGIdb database and published articles and considered them as drug agents for further analysis. Then, structural interactions between KGs and drug agents were performed and binding affinity scores (BAS) (kCal/mol) were calculated by molecular docking analysis. We presented the molecular docking results of the top-ranked interaction with BAS < −7 in Table 3. Five candidate drugs, namely, imatinib, regorafenib, pazopanib, teniposide, and dexrazoxane, are proposed for TNBC based on a threshold of BAS < −10 and highlighted by asterisks (*) in Table 3. The 3D (Three-dimensional) structural interactions of ligands (candidate drugs) and receptors (KGs) and their active sites are presented in Figure 8. The 2D (two-dimensional) structural binding of ligands and receptors and their interaction types are presented in Figure 9.

**TABLE 3:** List of drugs associated with corresponding targets, calculated binding affinity scores (BAS), and proposed candidate drugs highlighted by bold and star (*).

Target	Drug	Docking score	Drug	Docking score
EGFR	Imatinib*	−**11.7**	Ibrutinib	−8.2
	Regorafenib*	−**10.6**	Talazoparib	−8.2
	Irinotecan	−9.4	Leucovorin	−8.2
	Sorafenib	−9.4	Amlexanox	−8.1
	Lapatinib	−8.9	Neratinib	−8.1
	Sonidegib	−8.8	Vemurafenib	−8
	Etoposide	−8.8	Dacomitinib	−7.9
	Trastuzumab	−8.8	Bosutinib	−7.9
	Ponatinib	−8.7	Afatinib	−7.8
	Brigatinib	−8.4	Encorafenib	−7.8
	Dasatinib	−8.4	Gefitinib	−7.7
	Crizotinib	−8.3	Osimertinib	−7.6
	Dabrafenib	−8.3	Vandetanib	−7.5
	Pemetrexed	−8.3	Erlotinib	−7.2
	Trametinib	−8.2	Erdafitinib	−7.2
AURKB	Pazopanib*	−10.8	Sorafenib	−9.3
	Lapatinib	−9.8	Vandetanib	−8.7
	Dasatinib	−9.7	Erlotinib	−7.6
BIRC5	Imatinib*	−11.8	Tretinoin	−7.5
	Lapatinib	−9.8	Sulindac	−7.5
	Methotrexate	−9.5	Doxorubicin	−7.2
	Indomethacin	−8.4	Epirubicin	−7.1
	Flutamide	−7.9	Trastuzumab	−7.1
	Erlotinib	−7.8	Docetaxel	−7.1
	Romidepsin	−7.5	Vorinostat	−7
TOP2A	Teniposide*	−11.1	Idarubicin hydrochloride	−9.2
	Dexrazoxane*	−10.1	Etoposide phosphate	−9.2
	Doxorubicin hydrochloride	−9.8	Idarubicin	−9.2
	Daunorubicin citrate	−9.8	Amsacrine	−8.8
	Daunorubicin	−9.8	Etoposide	−8.8
	Doxorubicin	−9.2	Mitoxantrone	−7.7
	Epirubicin	−9.2	Podofilox	−7.6
ACTB	Ethinyl Estradiol	−8.1	Cyclophosphamide	−7.8

To validate the top-ranked drug molecules with some cancer-related PTM sites of top-ranked key proteins (EGFR, AURKB, BIRC5, and TOP2A) by their docking analysis, we predicted the their PTM sites (phosphorylation, succinylation, and ubiquitination) by the web-based prediction models MusiteDeep (https://www.musite.net/) and SuccinSite (http://systbio.cau.edu.cn/SuccinSite/). We found seven, seven, four, and six phosphorylated PTM sites in chain A and three, five, four, and six PTM sites in chain B for proteins EGFR, AURKB, BIRC5, and TOP2A, respectively (Supplementary File S6). Similarly, we found a total of 15 succinylated PTM sites in chain A and 11 PTM sites in chain B, and 11 ubiquitinated PTM sites in chain A and nine PTM sites in chain B **(**in Supplementary File S7,S8 **).** We observed that almost all active sites of blind docking are common with the predicted PTM sites (Figure 9). Furthermore, we performed the docking analysis between the predicted PTM sites and candidate drug molecules and observed their significant binding affinity scores (Table 4).

**TABLE 4:** Docking scores between the proposed drug molecules and the PTM sites of key proteins.

Drug molecule	Chain A							Chain B				
	Phosphorylated PTM sites of EGFR protein											
	S7	S63	S65	S162	T164	S168	S203	T93	Y95	T168		
Imatinib	-9.7	−7.9	−10.2	−6.8	−10.1	−6.7	−5.8	−8.3	−9	−8.1		
Regorafenib	−8	−7.8	−9.1	−13.1	−8.2	−7.4	−8.1	−7.8	−9.2	−7.1		
	Phosphorylated PTM sites of AURKB protein											
	S10	T12	T21	S170	S175	T180	S290	S1	T60	S61	S62	S67
Pazopanib	−10	−7.2	−7.1	−8.5	−12.1	−6.2	−5.6	−9.1	−8.2	−7.5	−11.1	−7.4
	Phosphorylated PTM sites of BIRC5 protein											
	S20	T34	T48	S81				S20	T34	T48	S81	
Imatinib	−7.3	−9.5	−8.2	−10.8				−7.3	−9.5	−8.2	−10.8	
	Phosphorylated PTM sites of TOP2A protein											
	S1	Y72	S148	S149	T215	S320	S1	Y72	S148	S149	T215	S320
Teniposide	−9.2	−7.8	−6.1	−10.8	−7.2	−9.4	−9.2	−7.8	−6.1	−10.8	−7.2	−9.4
Dexrazoxane	−8.6	−7.7	−6.4	−9.5	−8.2	−9.3	−8.6	−7.7	−6.4	−9.5	−8.2	−9.3
**Succinylated PTM sites of EGFR protein**												
**Drug molecule**	K42	K103	K142	K149				K65				
Imatinib	−9.2	−7.4	−9.6	−4.2				−8.3				
Regorafenib	−8.9	−7.1	−8.7	−5.3				−7.9				
Succinylated PTM sites of AURKB protein												
	K106	K239						K50				
Pazopanib	−7.2	−9.9						−5.3				
Succinylated PTM sites of BIRC5 protein												
	K15	K23	K103					K15	K23	K103		
Imatinib	−7.8	−8.6	−5.2					−7.8	−8.6	−5.2		
Succinylated PTM sites of TOP2A protein												
	K8	K55	K248	K306	K314	K357	K8	K55	K248	K306	K314	K357
Teniposide	−10.7	−9.2	−6.4	−9.2	−7.8	−8.1	−10.7	−9.2	−6.4	−9.2	−7.8	−8.1
Dexrazoxane	−9.9	−8.6	−6.3	−8.6	−8.8	−7.4	−9.9	−8.6	−6.3	−8.6	−8.8	−7.4
**Ubiquitinated PTM sites of EGFR protein**												
**Drug molecule**	K42	K103					K212					
Imatinib	8.3	7.4					9.8					
Regorafenib	7.9	7.3					10.1					
Ubiquitinated PTM sites of AURKB protein												
	K106	K254					K8					
Pazopanib	9.1	7.4					8.6					
Ubiquitinated PTM sites of BIRC5 protein												
	K62	K103	K115				K62	K103	K115			
Imatinib	8.2	12.3	7.1				8.2	12.3	7.1			
Ubiquitinated PTM sites of TOP2A protein												
	K68	K293	K306	K357			K68	K293	K306	K357		
Teniposide	8.3	6.7	11.8	12.7			8.3	6.7	11.8	12.7		
Dexrazoxane	7.9	7.1	10.9	12.6			7.9	7.1	10.9	12.6		

## Discussion

In this study, we identified 64 DEGs between 304 TNBC and 109 control samples (Supplementary File S2) by combining five transcriptomics datasets of NCBI with GEO accession numbers GSE65216, GSE76275, GSE38959, GSE27447, and GSE61724, and 306 DEGs between 116 TNBC and 124 control samples of RNA-Seq profiles from TCGA dataset (Supplementary File S3). Then, we constructed two sets of 10 top-ranked key-DEGs for both DEGs-sets by PPI network and their module analysis. We found seven common key-DEGs (TOP2A, BIRC5, EGFR, AURKB, ACTB, ASPM, and BUB1B) in both key DEGs-sets (Figures 3A,B **,** and Supplementary Figures S1A,B **)**, where six KGs (TOP2A, BIRC5, AURKB, ACTB, ASPM, and BUB1B) were upregulated and only one gene (EGFR) was downregulated (Table 2). Several studies have already proposed our identified KGs as TNBC-causing genes, which strongly supports our results. TOP2A encodes topoisomerase (DNA) II alpha, somatic mutations in a TOP2A immunohistochemical score may be important in predicting response to immunotherapy treatment for triple-negative breast cancer (Jiang et al., 2021). Several studies have proposed TOP2A as a prognostic marker and therapeutic target for TNBC through well-established integrated bioinformatics methods (Qiu et al., 2021; Wei et al., 2021; Ma et al., 2022). The gene expression of TOP2A and EGFR identifies efficacy of docetaxel plus epirubicin as neoadjuvant chemotherapy in TNBC patients (Liu et al., 2016). The EGFR gene encodes a receptor protein called the epidermal growth factor receptor, which causes cell proliferation, invasion, angiogenesis, and metastasis associated with poor prognosis in TNBC (Nakai et al., 2016). MicroRNA-203 may function to inhibit the proliferation and migration of TNBC cells by targeting BIRC5, so BIRC5 may be a potential therapeutic target for the treatment of TNBC patients (Wang et al., 2012). AURKB gene polymorphism can predict the overall survival or disease-free survival in TNBC patients treated with taxane-based adjuvant chemotherapy (Liao et al., 2018). The expression patterns of KGs with different subtypes of BC were significantly contrasted from the control group, where TNBC showed highly significant differences (Figure 5). The expression analysis of KGs with different BC progression stages compared to the normal stage indicated that the proposed KGs might be potential biomarkers for early diagnosis of TNBC (Supplementary Figure S2).

To investigate the pathogenetic mechanisms of TNBC with highlighting KGs, we performed GO functional and KEGG pathway enrichment analyses. We found some significant functional terms and pathways that are responsible for TNBC development which involves our identified KGs. The BP term mitotic spindle organization (involved in KG: BUB1B**,** AURKB, BIRC5, and TOP2A) was reported to be responsible for TNBC (Chimplee et al., 2022). Integrated bioinformatics analysis reported that microtubule cytoskeleton organization involved in mitosis (involved in KG: BUB1B**,** AURKB, and BIRC5) and kinase binding (involved in KG: AURKB, ACTB, EGFR, and TOP2A) are significant functional term for TNBC progression (Ryall et al., 2015; Huo et al., 2021). Therapeutic targets for TNBC treatment may interfere with the progression of TNBC by participating in the estrogen signaling pathway (involved in KG: EGFR, TOP2A, and BIRC5) (Huang et al., 2021). The regulatory interaction networks (miRNA-KG-TF) revealed three TFs proteins (SOX2, E2F4, and KDM5B) and three miRNAs (hsa-mir-1-3p, hsa-mir-124-3p, and hsa-mir-34a-5p) as the transcriptional and post-transcriptional regulators of KGs in Figure 7. The TF-protein, SOX2, has been found to be a tumor promoter and has a dynamic therapeutic strategy for TNBC (Liu et al., 2018). The expression of E2F4 is associated with lymph node metastasis in TNBC patients (Zou et al., 2022). The expression of KDM5B enhances invasive TNBC by downregulating hsa-mir-448 (Bamodu et al., 2016). Our identified three post-transcriptional regulators (hsa-mir-1-3p, hsa-mir-124-3p, and hsa-mir-34a-5p) are also supported by published articles that are associated with TNBC progression by using bioinformatics analysis and cellular experiments (Volovat et al., 2020; Zhang et al., 2021a; Zhang et al., 2021b; Shadbad et al., 2021).

**FIGURE 10:**
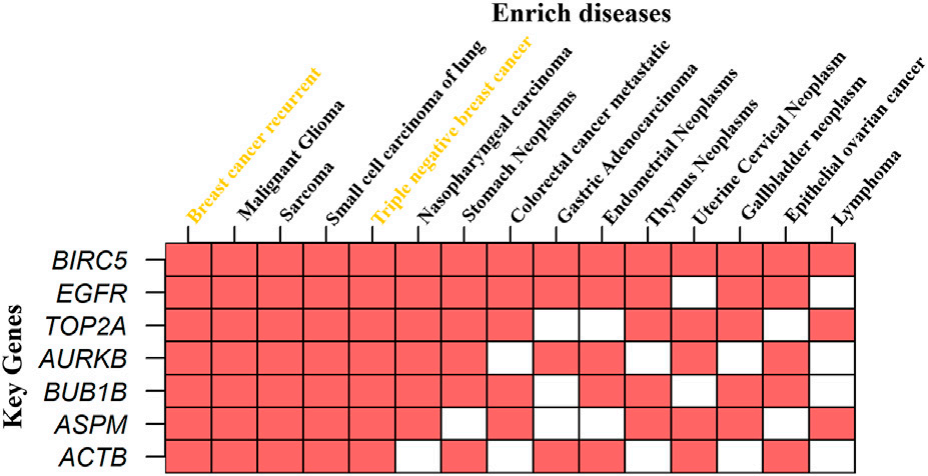
KG–disease interaction to check the enrichment of KGs in different diseases, where the *X*-axis represents diseases, the *Y*-axis represents KGs, and red indicates enriched.

We performed KG–disease interactions to check the enrichment of KGs in diseases from the DisGeNET database through the online based tool Enrichr (https://maayanlab.cloud/Enrichr/) (Chen et al., 2013), as shown in Figure 10. In the figure, we present the 15 most enriched diseases corresponding to our seven identified KGs. We observed that all KGs are highly enriched in recurrent BC, and one major subtype triple-negative BC highlighted in yellow strongly supports our proposed results. Another four diseases (malignant glioma, sarcoma, small cell carcinoma of lung, and nasopharyngeal carcinoma) also enriched all KGs, and one KG TOP2A enriched by all the top 15 diseases. Overall, the results suggested that our identified KGs may be significant for TNBC and some other diseases.

To discover the potential repurposable candidate drug molecules for the treatment against TNBC, 115 KG-associated FDA-approved drugs were collected from the DGIdb database and published articles. Among them, five drugs (imatinib, regorafenib, pazopanib, teniposide, and dexrazoxane) have been proposed as the candidate drug molecules for TNBC through the molecular docking analysis (Table 3). Then, we validated them by molecular docking analysis with some cancer-related PTM-sites (phosphorylation, succinylation, and ubiquitination) of the four top-ranked key proteins EGFR, AURKB, BIRC5, and TOP2A (Table 4). Imatinib was approved by the US Food and Drug Administration (FDA) on 1 February 2001 for the treatment of malignant metastatic and unresectable gastrointestinal stromal tumors (GISTs) (Dagher et al., 2002). Imatinib (also known as Glivec and Gleevec) is used to treat GISTs, acute lymphocytic leukemia (ALL), chronic myelogenous leukemia (CML), and other malignancies (Iqbal and Iqbal, 2014). It may be a novel treatment strategy for BC patients (including lethal subtype TNBC) overexpressing geminin and nuclear c-Abl (Blanchard et al., 2014). Regorafenib, sold under the brand names Stivarga and Regonix (in Bangladesh), was approved by the FDA in 2012 and 2013 for the treatment of metastatic colorectal cancer and GISTs, respectively (Regorafenib, 2017). Mehta et al. proposed that a combination of regorafenib, angiogenesis inhibitors, and radiation may be effective in inhibiting TNBC cells (Mehta et al., 2021). The drug pazopanib, also known as Votrient, was approved by the FDA on 19 October 2009, the EMA on 14 June 2010, the MHRA on 14 June 2010, and Australia’s TGA on 30 June 2010 for the treatment of advanced renal cell carcinoma and advanced soft tissue sarcomas (Bukowski et al., 2010; Nieto et al., 2011). It may play an important role in keeping the disease stable in advanced BC (under phase II clinical trial) and also TNBC (Taylor et al., 2010; Van Swearingen et al., 2017). Teniposide (known as Vumon), commonly used for the treatment of cancer diseases in children, was approved by the FDA for the treatment of second-line therapy of ALL in combination with other antineoplastic drugs (Drugs, 2007). Dexrazoxane was approved and designated by the FDA for the treatment of extravasation resulting from IV anthracycline chemotherapy and cardiomyopathy for children (0–16 years) and adults (FDA, 2013). Our five proposed candidate drugs are not approved for TNBC/BC yet, but several computational studies have suggested that they might be effective for TNBC/BC patients. This study lends further validation for the proposed candidate drugs and target proteins in the experimental lab as a proper treatment plan against TNBC.

## Conclusion

The selection of few potential target proteins and drug agents from a huge number of alternatives are equally important in drug discovery by wet-lab experiments. In this study, we identified triple-negative breast cancer (TNBC) causing seven KGs (TOP2A, BIRC5, AURKB, EGFR, ACTB, ASPM, and BUB1B) based on five NCBI-GEO microarray gene expression datasets and TCGA RNA-Seq datasets for early diagnosis and therapies of TNBC by using the integrated bioinformatics and systems biology approaches. We also confirmed their higher differential expression between normal and TNBC compared to other subtypes of BC from the other independent data source. The enrichment analysis of GO-terms and KEGG pathways with the key genes detected some crucial TNBC-related biological processes, molecular functions, cellular components, and pathways. For example, the detected mitotic spindle organization function and estrogen signaling pathway are both significantly associated with TNBC progression. The key gene (KG) regulatory network analysis detected some transcriptional and post-transcriptional regulators of KGs that are associated with TNBC progression. Finally, we proposed five KG-guided repurposable drug molecules (imatinib, regorafenib, pazopanib, teniposide, and dexrazoxane) for TNBC through network pharmacology and molecular docking analyses. Therefore, our findings would be more reliable for the wet-lab researchers and medical doctors in TNBC diagnosis and therapies at the earlier stage.

## Data Availability

The microarray gene expression datasets that analyzed in this study are available in the publicly accessible NCBI-GEO data repository as mentioned below: https://www.ncbi.nlm.nih.gov/geo/query/acc.cgi?acc=GSE65216, https://www.ncbi.nlm.nih.gov/geo/query/acc.cgi?acc=GSE76275, https://www.ncbi.nlm.nih.gov/geo/query/acc.cgi?acc=GSE38959, https://www.ncbi.nlm.nih.gov/geo/query/acc.cgi?acc=GSE27447, https://www.ncbi.nlm.nih.gov/geo/query/acc.cgi?acc=GSE61724. The PC related RNA-Seq profiles were downloaded from the TCGA data repository link https://portal.gdc.cancer.gov/projects.
