# Supplementary material for: Gene expression profile analysis to discover molecular signatures for early diagnosis and therapies of triple-negative breast cancer
Source: Front Mol Biosci. 2022 Dec 7;9:1049741. doi: 10.3389/fmolb.2022.1049741 (PMC9768339; doi:10.3389/fmolb.2022.1049741)
Supplement: Supplementary file 7 [file DataSheet1.DOCX]

**Gene expression profile analysis to discover molecular signatures for early diagnosis and therapies of triple negative breast cancer**

**Supplementary File S1**

| Contents | Pages |
| --- | --- |
| **Table S1:** List of TNBC-causing key common DEGs identified by different individual studies, where DEGs were calculated from each dataset separately. | 1-2 |
| **Table S2:** Top-ten enriched Gene ontology (GO) terms and KEGG pathways of DEGs by involving at-least one KGs. Highlighting KGs by bold. | 3-4 |
| **FIGURE S1.** Module analysis for both of NCBI-GEO and TCGA data (A) two cluster for DEGs of NCBI-GEO data and (B) top-two ranked cluster for DEGs of TCGA data. | 5 |
| **FIGURE S2.** Box plots for the expressions of KGs with different stages (Stage 1, Stage 2, Stage 3, and Stage 4) of BC including control group. | 6 |

| **Table S1:** List of TNBC-causing key common DEGs identified by different individual studies, where DEGs were calculated from each dataset separately. | | | |
| --- | --- | --- | --- |
| Author/PMID | Datasets ID (case/control) | Identified TNBC-causing key common DEGs | Suggested drug molecules |
| (Ma et al., 2022) | GSE65194 (41/11), GSE64790 (3/3), GSE41970 (160/40), and GSE38959 (30/13) | TOP2A, PCNA, MSH2, CDK6, CCNA2, MSH2, and CDK6 | Not Available (NA) |
| (Qiu et al., 2021) | GSE42568 (104/17), GSE45827 (41/11), and GSE76124 (11/14) | CCNB2, FOXM1, HMMR, MAD2L1, RRM2, TPX2, TYMS, CEP55, AURKA, CCNB1, CDK1, TOP2A, PBK | NA |
| (Yi et al., 2021) | GSE31519 (67) | CCR2 and CCR5 | NA |
| (Liu et al., 2021) | GSE62931 (47/53) and GSE76275 (198/67) | CDC20 | ZINC000004098930 and ZINC000004098930 |
| (He et al., 2021) | GSE26338 (66/5) | C1QB, CEP55, HIST1H2BO, IFI6, KIAA0101, PBK, SPAG5, SPP1, DCN, FZD7, KRT5, and TGFBR3 | NA |
| (Yuan et al., 2021) | GSE36693 (21/66) and GSE65216 (55/109) | CCNE1 | NA |
| (Xiao et al., 2021) | GSE38959 (30/13) and GSE65212 (41/11) | CCNB1, NCAPG, MCM4 and RRM2 | NA |
| (Li et al., 2021) | GSE27447 (5/14), GSE39004 (123/57), GSE43358 (17/40) and GSE45827 (41/11) | CD3D, CD3E, CD3G, FYN, GRAP2 and ITK | NA |
| (Jia et al., 2021) | GSE38959 (30/13) | MAPT, CBS, SOX11, IL6ST, and MEX3A | NA |
| (Ren et al., 2020) | GSE62931(47/53) | PLK1 | NA |
| (Lin et al., 2021) | GSE65194 (41/11) and GSE76124 (11/14) | RPS9, RPS14, RPS27, RPL11, and RPL14 | NA |
| (Zhai et al., 2020) | GSE45827 (41/11), GSE38959 (30/13), GSE65194 (41/11) | NUF2 and FAM83D | NA |
| (Chen et al., 2020) | GSE86945 (100/0), GSE86946 (58/0) and GSE102088 (0/114) | HSP90AA1, SRC, HSPA8, ESR1, ACTB, PPP2CA, and RPL4 | NA |
| (Fei et al., 2020) | GSE65194 (41/11), GSE43358 (17/40), GSE76275 (198/67) | NAT1, GATA3 and SCUBE2 | NA |
| (Lu et al., 2020) | GSE61724 (16/48), GSE64790 (3/3), GSE65194 (41/11) and GSE76250 (165/33) | CDK1, CCNB1, and CCNA2 | NA |
| (Chuan et al., 2020) | GSE53752 (51/25), GSE45827 (41/11), GSE65194 (41/11), and GSE38959 (30/13) | CXCR4 and CXCL10 | NA |
| (Zhai et al., 2019) | GSE76275 (198/67) | EGFR, KRT16, RET, SOX10, PDZK1, XBP1, TFF3, PTGER3, NME5, and IL6ST | NA |
| (Guo et al., 2017) | GSE27447 (5/14), GSE61724 (16/48), and GSE18864 (24/60) | GPR160, NAT1, AGR2, AGR3 and ERBB4 | NA |
| (Peng et al., 2017) | GSE41970 (160/40) | TP53, GAPDH, CCND1, HRAS and PCNA | NA |
| (He et al., 2015) | GSE18864 (24/60), and GSE27447 (5/14) | DUSP1, MYEOV2 and UQCRQ | NA |

| **Table S2:** Top-ten enriched Gene ontology (GO) terms and KEGG pathways of DEGs by involving at-least one KGs. Highlighting KGs by bold. | | |
| --- | --- | --- |
| Terms | P_Value | Involving DEGs |
| **GO_BP** | | |
| mitotic spindle organization (GO:0007052) | 2.4E-15 | RANBP2;CEP126;**BUB1B**;KIF23;TTK;**AURKB**;NDC80;TPX2;PRC1;NUF2;**BIRC5**;BUB1;DLGAP5;**TOP2A** |
| microtubule cytoskeleton organization involved in mitosis (GO:1902850) | 9.1E-12 | RANBP2;CEP126;NUF2;**BUB1B**;**BIRC5**;TTK;BUB1;DLGAP5;**AURKB**;NDC80 |
| regulation of protein kinase B signaling (GO:0051896) | 1.9E-08 | ITGB1;NTRK2;**BIRC5;**ERBB4;PTEN;FYN;PTPN11;LRP2;ESR1;**EGFR** |
| mitotic sister chromatid segregation (GO:0000070) | 3.5E-08 | PRC1;KIF14;NCAPG;**BIRC5;**KIF23;CEP55;DLGAP5;**AURKB**;NDC80 |
| regulation of cell population proliferation (GO:0042127) | 1.0E-07 | NTRK2;NOTCH1;STAT3;KIF14;PTEN;FN1;TTK;PTPRK;IGF1;RERG;**EGFR**;ERBB4;PRC1;**BIRC5** |
| positive regulation of cell population proliferation (GO:0008284) | 2.8E-07 | NTRK2;NOTCH1;ERBB4;PRC1;KIF14;PTEN;FN1;**BIRC5**;TTK;IGF1;**EGFR** |
| regulation of cell migration (GO:0030334) | 6.3E-07 | ITGB1;NOTCH1;**AURKB**;ERBB4;STAT3;KIF14;PTEN;PTPRK;IGF1;SULF1;**EGFR** |
| positive regulation of protein kinase B signaling (GO:0051897) | 8.1E-07 | ITGB1;ERBB4;**TOP2A**;FYN;PTPN11;LRP2;ESR1;**EGFR** |
| response to reactive oxygen species (GO:0000302) | 1.2E-06 | APOD;FYN;**BIRC5;**PTPRK;FOS;**EGFR** |
| mitotic spindle elongation (GO:0000022) | 1.7E-06 | PRC1;**BIRC5;**KIF23;**AURKB** |
| **GO_CC** | | |
| intracellular membrane-bounded organelle (GO:0043231) | 6.7E-08 | **TOP2A**;RPL4;NOTCH1;PTEN;ITPR1;NCAPG;MCM10;TTK;PTPRK;**ACTB**;RERG;**AURKB**;ARNTL2;**EGFR**;RAD51AP1;PTTG1;ERBB4;GRAP2;CMYA5;FYN;BUB1;DLGAP5;TP63;RANBP2;HSPA8;IL33;STAT3;KIF23;PTPN11;FOS;ESR1;NDC80;**ASPM**;TPX2;MELK;PRC1;**BIRC5** |
| nucleus (GO:0005634) | 8.3E-08 | **TOP2A**;RPL4;NOTCH1;PTEN;NCAPG;MCM10;TTK;**ACTB**;RERG;**AURKB**;ARNTL2;**EGFR**;RAD51AP1;PTTG1;ERBB4;GRAP2;CMYA5;FYN;DLGAP5;TP63;RANBP2;HSPA8;IL33;STAT3;KIF23;PTPN11;FOS;ESR1;NDC80;**ASPM**;TPX2;MELK;PRC1;**BIRC5** |
| spindle (GO:0005819) | 2.6E-06 | TPX2;PRC1;**BUB1B**;**BIRC5**;KIF23;TTK;**AURKB** |
| microtubule (GO:0005874) | 2.5E-05 | TPX2;PRC1;KIF14;**BIRC5**;KIF23;**AURKB** |
| intracellular non-membrane-bounded organelle (GO:0043232) | 6.3E-05 | **TOP2A**;RPL4;CDCA2;**BUB1B**;MCM10;KIF23;TTK;**ACTB**;**AURKB**;ARNTL2;RAD51AP1;TPX2;PRC1 |
| microtubule cytoskeleton (GO:0015630) | 8.8E-05 | TPX2;PRC1;KIF14;**EGFR**;**BUB1B**;KIF23;TTK;**AURKB** |
| chromosome (GO:0005694) | 1.6E-04 | **TOP2A**;RAD51AP1;CDCA2;**BIRC5**;NCAPG |
| membrane raft (GO:0045121) | 1.7E-04 | ITGB1;FYN; **BIRC5**;SULF1;**EGFR**;ABCG2 |
| glial cell projection (GO:0097386) | 8.9E-04 | ITGB1;FYN |
| spindle microtubule (GO:0005876) | 9.8E-04 | PRC1;**BIRC5**;**AURKB** |
| **GO_MF** | | |
| protein tyrosine kinase activity (GO:0004713) | 1.2E-06 | NTRK2;MELK;ERBB4;TTK;FYN;**EGFR** |
| kinase binding (GO:0019900) | 1.9E-06 | TPX2;PRC1;**TOP2A**;STAT3;KIF14;PTPN11;PTPRK;ESR1;**ACTB**;**AURKB**;**EGFR** |
| protein kinase binding (GO:0019901) | 4.3E-06 | **TOP2A**;ITGB1;TPX2;PRC1;STAT3;KIF14;PTPN11;PTPRK;ESR1;**ACTB** |
| tubulin binding (GO:0015631) | 5.5E-05 | TPX2;PRC1;KIF14;**BIRC5**;KIF23;FYN;DLGAP5 |
| microtubule binding (GO:0008017) | 9.7E-05 | TPX2;PRC1;KIF14;**BIRC5**;KIF23;DLGAP5 |
| protease binding (GO:0002020) | 5.5E-04 | ITGB1;NTRK2;PTEN;FN1 |
| transmembrane receptor protein kinase activity (GO:0019199) | 9.3E-04 | NTRK2;ERBB4;**EGFR** |
| transmembrane receptor protein tyrosine kinase activity (GO:0004714) | 9.3E-04 | NTRK2;ERBB4;**EGFR** |
| ATPase binding (GO:0051117) | 0.00165 | PGR;ESR1;**EGFR** |
| **KEGG** | | |
| Proteoglycans in cancer | 9.6E-10 | ITGB1;ERBB4;**AURKB**;STAT3;ITPR1;FN1;PTPN11;IGF1;ESR1;**ACTB**;**EGFR** |
| Estrogen signaling pathway | 2.72E-07 | HSPA8;**TOP2A**;**BIRC5**;ITPR1;PGR;TFF1;FOS;ESR1;**EGFR** |
| Breast cancer | 4.3E-07 | NOTCH1;PTEN;PGR;FOS;IGF1;ESR1;**EGFR** |
| Focal adhesion | 3.5E-06 | ITGB1;PTEN;**BIRC5**;FN1;FYN;IGF1;**ACTB**;**EGFR** |
| Pathways in cancer | 6.6E-06 | ITGB1;NOTCH1;STAT3;PTEN;FN1;**BIRC5**;FOS;IGF1;ESR1;**EGFR** |
| PD-L1 expression and PD-1 checkpoint pathway in cancer | 9.6E-06 | STAT3;PTEN;PTPN11;FOS;**EGFR** |
| Chemical carcinogenesis | 1.1E-04 | STAT3;**BIRC5**;PGR;FOS;ESR1;**EGFR** |
| PI3K-Akt signaling pathway | 1.3E-04 | ITGB1;NTRK2;ERBB4;PTEN;FN1;IGF1;**EGFR** |
| MAPK signaling pathway | 3.5E-04 | HSPA8;NTRK2;ERBB4;FOS;IGF1;**EGFR** |


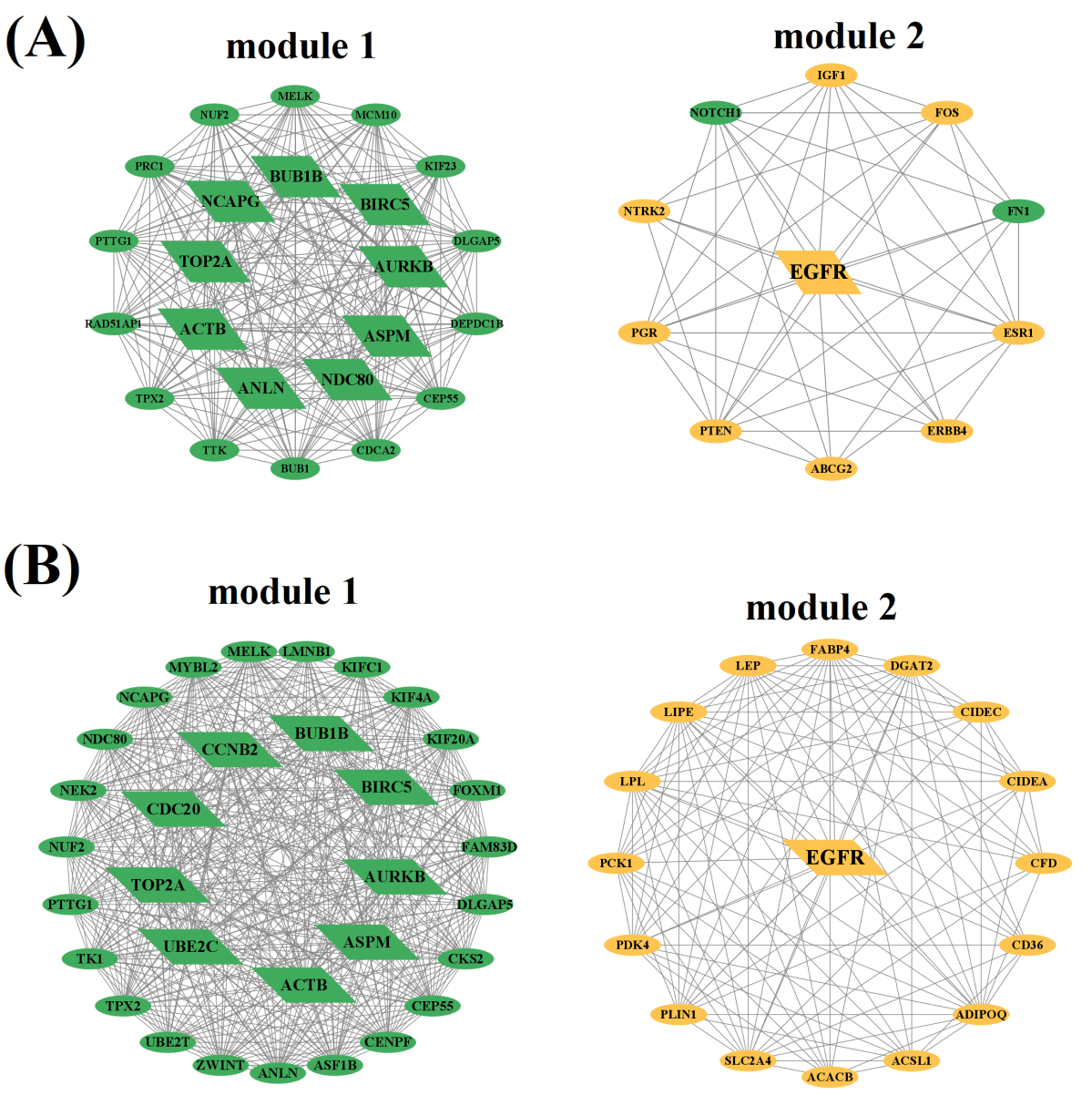


**FIGURE S1.** Module analysis for both of NCBI-GEO and TCGA data (A) two cluster for DEGs of NCBI-GEO data and (B) top-two ranked cluster for DEGs of TCGA data.

**
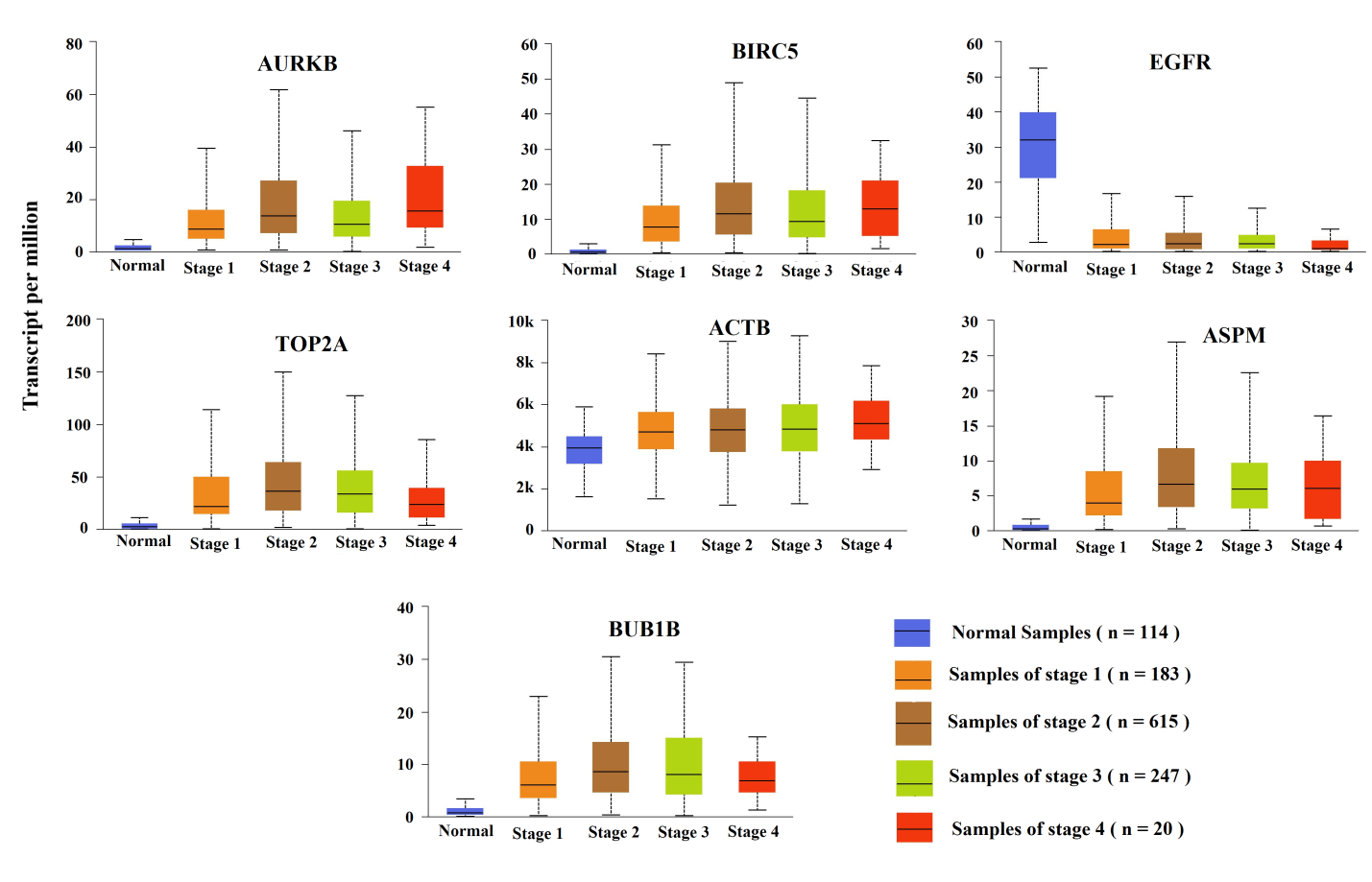
**

**FIGURE S2.** Box plots for the expressions of KGs with different stages (Stage 1, Stage 2, Stage 3, and Stage 4) of BC including control group.

**References:**

Chen, J., Liu, C., Cen, J., Liang, T., Xue, J., Zeng, H., et al. (2020). KEGG-expressed genes and pathways in triple negative breast cancer: Protocol for a systematic review and data mining. *Medicine (Baltimore)* 99, e19986. doi: 10.1097/MD.0000000000019986.

Chuan, T., Li, T., and Yi, C. (2020). Identification of CXCR4 and CXCL10 as Potential Predictive Biomarkers in Triple Negative Breast Cancer (TNBC). *Med Sci Monit* 26, e918281. doi: 10.12659/MSM.918281.

Fei, H., Chen, S., and Xu, C. (2020). RNA-sequencing and microarray data mining revealing: the aberrantly expressed mRNAs were related with a poor outcome in the triple negative breast cancer patients. *Ann Transl Med* 8, 363. doi: 10.21037/atm.2020.02.51.

Guo, J., Gong, G., and Zhang, B. (2017). Screening and identification of potential biomarkers in triple-negative breast cancer by integrated analysis. *Oncol Rep* 38, 2219-2228. doi: 10.3892/or.2017.5911.

He, J., Yang, J., Chen, W., Wu, H., Yuan, Z., Wang, K., et al. (2015). Molecular Features of Triple Negative Breast Cancer: Microarray Evidence and Further Integrated Analysis. *PLoS One* 10, e0129842. doi: 10.1371/journal.pone.0129842.

He, Y., Cao, Y., Wang, X., Jisiguleng, W., Tao, M., Liu, J., et al. (2021). Identification of Hub Genes to Regulate Breast Cancer Spinal Metastases by Bioinformatics Analyses. *Comput Math Methods Med* 2021, 5548918. doi: 10.1155/2021/5548918.

Jia, R., Weng, Y., Li, Z., Liang, W., Ji, Y., Liang, Y., et al. (2021). Bioinformatics Analysis Identifies IL6ST as a Potential Tumor Suppressor Gene for Triple-Negative Breast Cancer. *Reprod Sci* 28, 2331-2341. doi: 10.1007/s43032-021-00509-2.

Li, L., Huang, H., Zhu, M., and Wu, J. (2021). Identification of Hub Genes and Pathways of Triple Negative Breast Cancer by Expression Profiles Analysis. *Cancer Manag Res* 13, 2095-2104. doi: 10.2147/CMAR.S295951.

Lin, Z., Peng, R., Sun, Y., Zhang, L., and Zhang, Z. (2021). Identification of ribosomal protein family in triple-negative breast cancer by bioinformatics analysis. *Biosci Rep* 41. doi: 10.1042/BSR20200869.

Liu, N., Wang, X., Zhu, Z., Li, D., Lv, X., Chen, Y., et al. (2021). Selected ideal natural ligand against TNBC by inhibiting CDC20, using bioinformatics and molecular biology. *Aging (Albany NY)* 13, 23702-23725. doi: 10.18632/aging.203642.

Lu, Y., Yang, G., Xiao, Y., Zhang, T., Su, F., Chang, R., et al. (2020). Upregulated cyclins may be novel genes for triple-negative breast cancer based on bioinformatic analysis. *Breast Cancer* 27, 903-911. doi: 10.1007/s12282-020-01086-z.

Ma, J., Chen, C., Liu, S., Ji, J., Wu, D., Huang, P., et al. (2022). Identification of a five genes prognosis signature for triple-negative breast cancer using multi-omics methods and bioinformatics analysis. *Cancer Gene Ther*. doi: 10.1038/s41417-022-00473-2.

Peng, C., Ma, W., Xia, W., and Zheng, W. (2017). Integrated analysis of differentially expressed genes and pathways in triplenegative breast cancer. *Mol Med Rep* 15, 1087-1094. doi: 10.3892/mmr.2017.6101.

Qiu, P., Guo, Q., Yao, Q., Chen, J., and Lin, J. (2021). Hsa-mir-3163 and CCNB1 may be potential biomarkers and therapeutic targets for androgen receptor positive triple-negative breast cancer. *PLoS One* 16, e0254283. doi: 10.1371/journal.pone.0254283.

Ren, Y., Deng, R., Zhang, Q., Li, J., Han, B., and Ye, P. (2020). Bioinformatics analysis of key genes in triple negative breast cancer and validation of oncogene PLK1. *Ann Transl Med* 8, 1637. doi: 10.21037/atm-20-6873.

Xiao, X., Zhang, Z., Luo, R., Peng, R., Sun, Y., Wang, J., et al. (2021). Identification of potential oncogenes in triple-negative breast cancer based on bioinformatics analyses. *Oncol Lett* 21, 363. doi: 10.3892/ol.2021.12624.

Yi, J., Zhong, W., Wu, H., Feng, J., Zouxu, X., Huang, X., et al. (2021). Identification of Key Genes Affecting the Tumor Microenvironment and Prognosis of Triple-Negative Breast Cancer. *Front Oncol* 11, 746058. doi: 10.3389/fonc.2021.746058.

Yuan, Q., Zheng, L., Liao, Y., and Wu, G. (2021). Overexpression of CCNE1 confers a poorer prognosis in triple-negative breast cancer identified by bioinformatic analysis. *World J Surg Oncol* 19, 86. doi: 10.1186/s12957-021-02200-x.

Zhai, Q., Li, H., Sun, L., Yuan, Y., and Wang, X. (2019). Identification of differentially expressed genes between triple and non-triple-negative breast cancer using bioinformatics analysis. *Breast Cancer* 26, 784-791. doi: 10.1007/s12282-019-00988-x.

Zhai, X., Yang, Z., Liu, X., Dong, Z., and Zhou, D. (2020). Identification of NUF2 and FAM83D as potential biomarkers in triple-negative breast cancer. *PeerJ* 8, e9975. doi: 10.7717/peerj.9975.
